# The Workplace and Alcohol Problem Prevention

**Published:** 2002

**Authors:** Paul M. Roman, Terry C. Blum

**Affiliations:** Paul M. Roman, Ph.D., is a distinguished research professor of sociology and director of the Center for Research on Behavioral Health and Human Services Delivery, University of Georgia, Athens, Georgia. Terry C. Blum, Ph.D., is dean and Tedd Munchak Professor at the DuPree College of Management, Georgia Institute of Technology, Atlanta, Georgia

**Keywords:** workplace-based prevention, Employee Assistance Program, intervention referral, relapse prevention, alcohol or other drug (AOD) education, health promotion, workplace AOD policy, identification and screening for AOD use, occupational stress, stress as an AOD cause (AODC), social detachment

## Abstract

Workplace programs to prevent and reduce alcohol-related problems among employees have considerable potential. For example, because employees spend a lot of time at work, coworkers and supervisors may have the opportunity to notice a developing alcohol problem. In addition, employers can use their influence to motivate employees to get help for an alcohol problem. Many employers offer employee assistance programs (EAPs) as well as educational programs to reduce employees’ alcohol problems. However, several risk factors for alcohol problems exist in the workplace domain. Further research is needed to develop strategies to reduce these risk factors.

As a domain for alcohol-problem prevention, the workplace holds great promise. In the United States and, increasingly, around the world, the majority of adults who are at risk for alcohol problems are employed. As described here, employers have several well-defined means at their disposal for intervening with problem drinking. Those methods serve not only the interests of the employer but also those of the employees and their dependents. Furthermore, the potential for a preventive impact is worldwide. Western styles of workplace organization and employment relationships have spread to influence global practices, setting the stage for the diffusion of workplace interventions and for addressing emerging economies’ increasing alcohol problems (Masi 2000; Roman in press).

Despite these possibilities, the development of prevention programs in U.S. workplaces has slowed considerably in recent years and, in fact, may be in need of revitalization (Roman and Baker 2001; Roman in press). The decline in work-place attention to alcohol problems illustrates the need for creating and maintaining an infrastructure for sustaining alcohol interventions in settings not typically associated with the delivery of health care.

This article will first review the opportunities workplaces provide for preventing alcohol problems—people spend a large amount of time at the workplace and employers may use their leverage to motivate an employee to seek help for an alcohol problem. The article also will discuss the use of employee assistance programs (EAPs) and complementary programs to reduce employee alcohol problems and then examine risk factors for alcohol problems that exist in the work environment.

## Tracing the Development of Workplace Programs

The significant presence of alcohol problems in the workforce was most recently documented in a 1997 national survey, indicating that about 7.6 percent of full-time employees are heavy drinkers (i.e., they consumed five or more drinks per occasion on 5 or more days in the month prior to being surveyed) (Zhang et al. 1999). According to that study, about one-third of the heavy drinkers also used illegal drugs.

Workplaces have introduced programs to prevent and treat alcohol and other drug (AOD) abuse among employees, especially over the past 25 years. The goal of many of these programs has been “human resource conservation”; that is, the programs strive to ensure that employees maintain their careers and productivity (Roman and Blum 1999). Although the programs vary considerably in their structure, they may include health promotion, education, and referral to AOD abuse treatment when needed. Most of these programs focus on early identification of a problem or helping those already affected by a problem (i.e., secondary prevention) rather than targeting the general population (i.e., primary prevention). Three separate studies show that the majority of American employers offer EAPs, which potentially may provide services to help eliminate drinking in the workplace (Zhang et al. 1999; Hartwell et al. 1996; Blum and Roman 1995). Despite the widespread use of such programs, however, no data from a representative sample of EAPs are available to support the usefulness of these programs.

## Opportunities for Workplace Prevention

The workplace provides several potent opportunities for implementing AOD abuse prevention strategies, including:

- The majority of adults are employed, making the workplace an ideal setting to reach a large population.
- Full-time employees spend a significant proportion of their time at work, increasing the possibility of exposure to preventive messages or programs offered through the work-place. The likelihood that evidence of problem drinking will become visible to those who might have a role in intervention also is increased.
- Work plays an important role in most people’s lives. Because many adults’ roles in the family and community are dependent on maintaining the income, status, and prestige that accompanies employment, the relationship between the employer and the employee contains a degree of “leverage.” The employer has the right to expect an adequate level of job performance. If alcohol abuse breaches the rules of the employer-employee agreement or is associated with substandard job performance, the employer may withdraw pay or privileges associated with the job, thus motivating the employee with alcohol problems to change his or her behavior.

## Primary and Secondary Prevention in the Workplace

Workplace programs include both primary and secondary prevention. Primary prevention aims to keep alcohol problems from developing, and secondary prevention seeks to reduce existing problems. Researchers have voiced concerns that workplace programs overemphasize secondary prevention (Ames and Janes 1992). Primary prevention often is more cost-effective than secondary prevention; however, the work-place is not conducive to strategies aimed at preventing alcohol use. Most employees are adults and therefore are legally allowed to consume alcohol. Employers rarely are in a position to prevent their employees from initiating drinking as an off-the-job lifestyle practice, nor do they desire to do so.

At the same time, employers want their employees to perform their jobs well and not disrupt or endanger coworkers’ activities. Smooth work transactions with customers and other members of the public also are important in many organizations, including the service sector.

Alcohol problems in the workplace are identified by these two, or sometimes three, events:

1. The linkage of a drinking pattern with job performance problems, such as a pattern of poor-quality work, poor quantity of work, attendance problems, or problems related to interaction with clients or customers.
2. Employees’ decisions that their drinking behaviors are causing problems for themselves and they desire assistance, leading to a self-referral to a source of assistance in the workplace.
3. In some settings, a coworker’s identification of an apparent alcohol problem is used to refer an employee for workplace-based assistance. This is the primary approach used in Member Assistance Programs, which have developed in some labor union settings (Bacharach et al. 1996).

## EAPS: Addressing Employees’ Alcohol Problems

EAPs are the most common intervention used in the workplace to address alcohol problems. EAPs have distinctive features that set them apart from prevention strategies used in other settings. Their goal is to prevent loss of employment and to assure that employed people continue their careers and productivity without interruption. EAPs can thus prevent both employer and the employee from suffering the costly consequences of the employee’s job loss.

### EAP Referral Routes and the EAP Process

#### Self-Referrals

Early in the development of the EAP model, researchers proposed that such programs would ideally operate by primarily attracting self-referrals rather than “coerced” referrals (Wrich 1973). Given that denial and resistance are common barriers to alcohol treatment, this was an unusual idea. Wrich (1973) claimed that significant rates of self-referral would increase the program’s credibility by demonstrating “consumer confidence.” In contrast, a program centered on supervisory referrals, which may or may not involve coercive pressure to use EAP services, implies a “correctional” image for the EAP.

On the surface, this ideal appears to have been achieved. Nearly all reports generated about EAP usage indicate a predominance of self-referrals. In those relatively rare instances where EAP referral processes have been examined in depth, the vast majority of cases are classified upon entry as “self-referrals” (Blum et al. 1995). However, these self-referrals may actually reflect cases in which employees were prompted by others to seek EAP assistance (described as “informal referrals” below). One study (Blum et al. 1995) found that only 18 percent of male and 22 percent of female referrals to EAPs with alcohol problems were “genuine” self-referrals—that is, those people reported it was their personal decision that drove them to seek help (Blum et al. 1995). Most of these employees reported few job problems. Through confidential questionnaires, they reported that the following three features of service access were essential in their decisions to seek help: (1) a professionally competent source of assistance was available for a range of personal difficulties, including alcohol problems; (2) service was provided by the employer; and (3) employees could use the service with assurance of confidentiality and without penalty to any aspect of their job status.

#### Informal Referrals

Another route to consulting EAPs is through informal referrals. In such cases, the referral is prompted by considerable social interaction and discussion, often involving an employee’s supervisor. Most of the referral processes are informal—about 80 percent of alcohol-problem referrals (self-referrals are included in this group)—and 20 percent are formal supervisory referrals (Blum et al. 1995). Although EAPs were originally designed as mechanisms for formal supervisory referral of problem employees, these figures show that they were quickly transformed into sources of help that people reached without going through explicitly formal channels.

For reasons that are largely self-evident, both supervisors and subordinates prefer these informal procedures. The disadvantage of the informal referral is that there is no official record of the employee being referred to the EAP or of any related job performance problems.

#### Formal Referrals

When external intervention is required, formal referrals are used. Such cases are prompted by a supervisor detecting a decline in job performance that cannot be explained by the conditions of work. Supervisors are urged to consult with EAP staff before taking action to assure that they are conforming to workplace policy. Procedures call for the supervisor to constructively confront employees if they deny their performance problems or are not willing to take corrective action. In such a confrontation, the supervisor presents evidence of the employee’s performance problems and points out that disciplinary measures will ensue if the problems are not corrected. A referral to the EAP is offered as a means for problem correction.

Should the employee elect to use the company program, the EAP coordinator conducts an assessment or arranges for a diagnosis of the employee’s problem. The coordinator or diagnostic agent then offers advice as to how the problem might be handled. Counseling or treatment at a community agency follows, with arrangements usually made by the EAP coordinator to assure the best match between quality of care and financial coverage available through the workplace.

It is important to emphasize that the use of treatment or counseling is a decision made by the employee and not a mandate from the employer. The employee is responsible for payment for services that the company’s health plan does not cover.

### The EAP’s Role in Followup and Relapse Prevention

After using EAP services and receiving counseling and treatment, the employee should ideally go through a period when his or her symptoms are in remission. However, relapse during the posttreatment period is very common for those with AOD problems. These relapses may account for what many regard as the disappointing overall success rates of alcohol-problem treatment and may have little or nothing to do with the quality of EAP services provided.

Relapse prevention encompasses a different range of interventions. Researchers often disregard it as a form of alcohol-abuse prevention. In many respects, the recovering person is set on a pathway of starting over, and it seems reasonable to conceptualize the prevention of relapse as primary prevention of the alcohol problem. Treatment programs vary greatly in the extent to which such services are provided after treatment ends. EAPs and workplaces can play important roles in relapse prevention, however. Opportunities for relapse prevention lie in the nature of work and access to employees who are attempting to maintain recovery. Unlike the community setting, where followup requires finding clients and/or motivating them to return to the treatment setting for aftercare counseling, the workplace has built-in opportunities to reach these persons and provide counseling and support necessary to sustain recovery. And it is also easier for the recovering employee to seek assistance, as needed, to assure recovery gains. Such an opportunity might not apply in the instance of an employee who had recovered from an alcohol problem prior to employment and did not desire to reveal this fact to a new employer.

Many EAPs include followup and relapse prevention to help employees maintain recovery. Only one research study, however, has systematically investigated the impact of such services. In that study, Foote and Erfurt (1991) examined the effects of posttreatment followup contact among a group of 164 EAP clients treated for alcohol problems over a period of 1 year. The tendency to relapse was significantly lower in the followup group, compared with a group of 161 similar clients who did not receive followup contact, indicating the efficacy of followup for relapse prevention.

### EAP Effectiveness and Maximizing EAP Use

A review (Blum and Roman 1995) of a wide range of published and unpublished evaluation research concludes that EAPs produce far more in savings than they require in costs. A series of evaluation studies indicated that the programs succeeded in returning substantial proportions of employees with alcohol problems to effective performance (Asma et al. 1980; Edwards et al. 1973; Eggum et al. 1980; Flynn et al. 1993; Gam et al. 1983; McAllister 1993; Spickard and Tucker 1984; Walsh et al. 1991, 1992). Most of the research supporting this conclusion has methodological limitations, however. None of the studies involved rigorous comparisons with settings where no EAP services were available. In addition, by examining clinical or performance outcomes among employees who have received treatment or counseling via EAP case management (which often includes followup), it is not possible to separate the effects of EAP services from other aspects of the referral-and-treatment process.

How can EAP utilization be maximized? Three published studies (Googins and Kurtz 1981; Hoffman and Roman 1984; Colan and Schneider 1992), differing in design and methods, reached the common conclusion that supervisory training significantly increased positive attitudes toward EAPs, increased the perceived likelihood of utilizing the service, and actually produced greater service utilization. The impact of training deteriorated over time, as would be expected, indicating the need for ongoing and repeated “boosters” to sustain attention to the service.

## Complements to EAPS

Because off-the-job drinking can affect worker performance and health but not necessarily reflect an alcohol problem that would result in an EAP referral, some employers offer programs to complement an existing EAP. Such programs are designed to educate employees about the potential effects of drinking and to encourage employees to seek help from an EAP when needed.

Epidemiological data cited earlier (Zhang et al. 1999) indicate that many employed people drink heavily or engage in binge drinking when they are away from work, leading to a variety of adverse consequences and problems (Calahan and Room 1974). Employers have valid reasons for motivating these employees to change their drinking patterns, as this type of problem drinking likely will have an impact on the workplace, although not necessarily in ways that are visible or even measurable.

Several recent studies have addressed the effects of hangovers on work performance. Hangovers affect cognitive and motor functions, creating risks of bad judgment, interpersonal conflict, and injuries (Moore 1998). Using observational and questionnaire data in an on-site study, Ames and colleagues (1997) concluded that hangovers are a significant contributor to job performance problems, yet discussions of alcohol’s impact on the workplace rarely recognize the costs of hangovers. Combining survey and observational techniques at multiple corporate sites, Mangione and colleagues (1999) reached similar conclusions about the hidden and subtle impact of hangovers on work performance.

As Moore (1998) pointed out, hangovers are clearly alcohol-related problems in the workplace but are extremely difficult to address through specific interventions because people define hangover differently. Mangione and colleagues (1999) suggested that employee education and corporate policy materials should include information about the potentially adverse effects of off-the-job drinking on workplace behavior and job performance.

### Alcohol Education Programs

The principal means for addressing an employee’s off-the-job drinking is through alcohol education programs conducted at the worksite. These programs usually are associated with an EAP, a health promotion program, or both. The goal of these education programs often is to encourage behavioral change or use of the associated services (i.e., self-referral to an EAP).

Several studies have examined the impact of alcohol education. In an early study, McLatchie and colleagues (1981), using 90- and 30-minute training sessions with supervisors and with employees, respectively, found significant changes in alcohol attitudes immediately following the sessions. Brochu and Souliere (1988) examined the impact of a “life skills re-education program” on changing new employees’ attitudes toward AODs. Although the study found significant effects of the program based on data collected immediately and after 1 month, followup at 36 months indicated no sustained effects.

A similar study by Kishchuk and colleagues (1994) tested a program designed to make employees’ drinking behaviors healthier and more socially responsible. Followup data collected 1 month later revealed modest impacts on attitudes and behavior. A placebo treatment providing nutrition education delivered to a comparison group also produced modest but significant changes in drinking, leading to the suggestion that the experience of training rather than its content may have notable importance. Another study evaluated a comprehensive approach to altering people’s drinking behavior as well as workplace culture in the 3M Company (Stoltzfus and Benson 1994). This program included a 10-hour supervisory training section, a 2.5-hour section for employees to discuss policies and their behavior, and a peer helper section. The pilot program was conducted at a Midwestern site matched with a comparison plant. Results showed that participants had lower alcohol consumption, lower incidence of work performance negatively affected by AOD use, and improved prevention skills.

In a similar study, Cook and colleagues (1996*a*) field-tested the Working People Program with 108 employees. The four-session training program significantly affected self-reported alcohol consumption and motivated employees to reduce consumption and the problem consequences of drinking. In another study of 371 employees randomly assigned to experimental and control groups, Cook and colleagues (1996*b*) evaluated the effects of three classroom sessions that used videos and booklets about AOD issues. Results from this study also indicated a significant increase in the motivation to reduce alcohol use among the group receiving the training.

The studies described here generally reported beneficial effects of workplace-based education on drinking behavior. This research has certain limitations, however. None of the studies replicates earlier findings; that is, each study stands alone. Further, the effects of the training usually were measured immediately or shortly after the sessions ended. In the one study with a longer followup period, the positive effects deteriorated completely (Brochu and Souliere 1988). Overall, three suggestions come from this research. First, alcohol education appears to be a useful investment, showing significant effects in all reported studies. Second, the data suggest that these effects need boosters if they are to be sustained, a finding common to most educational interventions. Third, it is clear that more research is needed to specify the training content required to improve efficacy and the durability of effects.

### Health Promotion Programs

In addition to alcohol education programs, employers also may offer health promotion programs, which may motivate employees to alter their drinking behaviors. When health problems such as weight, high blood pressure, or gastric problems are identified in a health risk survey administered at the work-site, the administering health worker may suggest a reduction in drinking as a means of alleviating the primary symptom. Alternatively, employees undertaking exercise programs or other health-oriented activities might change their drinking behavior because drinking may not be consistent with their new healthy regimen.

Research on the impact of workplace health promotion programs on employee drinking is sparse. Shain and colleagues (1986) collected short-term evaluative data in several Canadian settings indicating that health promotion and wellness programs can significantly reduce employee drinking. In particular, the authors state that heavy drinkers are characterized by a series of unhealthy behaviors that can be addressed through a wellness program. Further, Shain and colleagues (1986) observe that healthy lifestyles and alcohol abuse are incompatible. They contend that the nesting of alcohol issues within larger health concerns is a highly effective means of motivating behavioral change toward less risky drinking and a healthier lifestyle in general.

### Peer Intervention

As deviant drinking patterns become more chronic and pervasive in an employed person’s life, his or her job performance will eventually be affected. Coworkers may notice job performance problems before such problems become evident to supervisors.

Employee alcohol education programs may prepare peers to suggest assistance to one another, but this has not been documented. More specifically, the techniques of peer intervention programs may be useful for addressing early problem behaviors, as has been documented among unionized workers (Bacharach et al. 1996). Peer intervention is not applicable in all settings, only where it is possible to tap into what Bacharach and his colleagues call “communal voluntarism,” or a committed desire of workers to look out for each other’s well-being.

Peer-assistance programs have been implemented among professional groups such as physicians, dentists, psychologists, attorneys, and airline pilots. Little is known about the operation of these interventions among professionals because they are conducted with high levels of confidentiality. Research has been conducted, however, on union-based Member Assistance Programs (Bacharach et al. 1994; Bamberger and Sonnenstuhl 1995). These programs are reported to be highly effective, although the extent to which they may provide early identification of alcohol problem behaviors has not been documented.

The programs described in this section primarily address the effects of off-the-job drinking and are designed to educate and aid employees. Participation in such programs is almost always voluntary. A considerably different employer attitude is found toward on-the-job drinking, which in most settings has been prohibited for many decades. Because drinking on the job can jeopardize the safety of the employee, the workplace, and the public, workplace alcohol policies are designed to set clear limits on alcohol use and establish consequences for employees who do not observe these limits.

## Workplace Policies Regarding Drinking on the Job and Alcohol Testing

As part of workplaces’ “rules of conduct” or “fitness for duty” regulations, supervisors are often empowered to discipline or remove an employee from the job on the suspicion of drinking. However, if an employee is suspected of drinking based on evidence such as odor of alcohol or appearance of intoxication, the employee may object, which could lead to litigation. When alcohol use is suspected, alcohol testing can be used to establish whether the employee was in fact drinking. Specific techniques include both breath testing and blood testing.

Macdonald (1997) asserts that alcohol testing is important in the work-place because drinking is distinctively linked to performance impairment, particularly when compared with other drugs. Alcohol testing is currently mandated for the transportation industry through Federal regulations. Alcohol testing is most commonly used in other workplace settings when cause is established, particularly in response to on-the-job accidents. In such cases, alcohol testing is critical in establishing possible culpability, especially if injuries have occurred. When alcohol tests are positive, case dispositions may vary according to company policy, ranging from dismissal to the offering of counseling or treatment under the auspices of an EAP. These actions appear to have substantial employee support. In a multisite survey of 6,540 employees, 81 percent were in favor of alcohol testing following a workplace accident, and 49 percent indicated support for random alcohol testing in the workplace (Howland et al. 1996).

## Risk Factors in the Work Environment

Compared with EAPs, prevention efforts focused on reducing risk factors in the work environment may offer the greatest potential payoff. This approach is the most problematic in terms of implementation, however. One possible avenue would be to identify and alter work environments that have “toxic” connections to alcohol problems. Employers would be reluctant, however, to participate in efforts that might highlight their liability in creating high-risk environments.

Despite the potential problems in implementing interventions to reduce risk factors in the workplace, research has examined several work-related factors that may contribute to alcohol use and related problems among employees. These risk factors are described below.

### Stress

Many studies have found significant but relatively small associations between stress in the workplace and elevated levels of alcohol consumption. For example, in one early study using survey data, Fennell and colleagues (1981) reported that employees’ reasons for drinking were found to be associated with stress-inducing job characteristics, but the correlations were relatively weak. In a national survey of employed persons, Martin and Roman (1996) found that lower job satisfaction and higher job stress both were risks for increased drinking. Lehman and colleagues (1995) reported significant associations between employee AOD use and lower job satisfaction, less faith in management, and lower involvement with and commitment to the job. Parker and Farmer (1990) reported significant associations between drinking and job burnout. Greenberg and Grunberg (1995) found negative associations between employee drinking behavior and reported job autonomy and job satisfaction.

Although this research may suggest certain preventive interventions, such as reducing work-related stress and increasing job satisfaction, it is unclear how to implement such changes. For example, although some workers may apparently drink less if their job satisfaction is enhanced, there are multiple sources of job satisfaction, some related to the job and others to a combination of a person’s background and his or her job characteristics. In addition, the direction of the relationships between stress or job dissatisfaction and drinking is unknown. For example, drinking and other drug use could contribute to the reports of work stress found in these studies. That is, employees experiencing the ongoing detrimental effects of off-the-job drinking may have greater difficulty in coping with “normal” workplace pressures.

Thus, to date, research has not yielded enough compelling evidence to guide the creation of workplace programs targeting work-related stress and job dissatisfaction with the goal of reducing alcohol problems. More research is necessary to specify the stress-drinking linkage and to identify the characteristics of workers most likely to be at risk for stress-related drinking. Such research also needs to examine the costs and benefits to employers of implementing changes that would influence worker stress, job satisfaction, and drinking.

### Alienation

Whereas work stress may be temporary, worker alienation is a considerably more pervasive and problematic risk factor among employed persons. Alienation relates to the employee’s broader sense of identity and control and has considerable implications for overall mental well-being. Seeman and colleagues (Seeman and Anderson 1983; Seeman et al. 1988) reported strong associations between alienation from work and employees’ drinking behavior, although others (Blum 1984; Parker and Farmer 1990) have challenged the methodology of their work. Lehman and colleagues (1995) also found an association between employee AOD use and estrangement or alienation from the job. In another study that focused on interpersonal conflict in the workplace, Rospenda and colleagues (2000) reported that “generalized workplace abuse” from supervisors or work peers was positively associated with increased drinking.

Although the above studies reported statistically significant findings, the reported relationships between work-place alienation and employee drinking are not powerful. As in the case of work stress, the direction of the relationship must be considered. For instance, problem drinkers have been shown to have impaired social relationships, which may contribute to alienation in the workplace.

Several emergent managerial strategies may directly address employee alienation and, in turn, influence the drinking that may be associated with alienation. These strategies are encompassed under the broad rubric of “participative management.” This approach, which calls for the involvement of employees in planning and decision-making about their work, is not predicated on reducing employee alienation but on enhancing their involvement, interest, and productivity. Reducing worker alienation may be an unanticipated side-effect. Participative management should not be viewed generically, for its implementation can vary greatly. One study (Barker 1993) found evidence to strongly suggest that under some conditions, participative management may create or escalate the very types of stress that have been linked with increased employee drinking in other research.

### Cultures and Subcultures

Worksites’ cultures and subcultures may have differential effects on encouraging or discouraging drinking and substance abuse. Cosper (1979) introduced the concepts that occupations have widely variant drinking norms associated with their cultures and that workers are differentially socialized into drinking according to their occupational choices. These concepts are augmented by the notion that heavy-drinking occupations attract job seekers who are prone to these behaviors, which is suggested, for example, by survey results that show high rates of heavy drinking among bartenders and restaurant workers as compared with other employed persons (Hoffman et al. 1997).

Clearly these drinking norms are differentially introduced into the occupational mixes found in workplaces. Ames and Delaney (1992) studied a large manufacturing plant in which on-the-job drinking and other drug use were unexpectedly prevalent. They viewed these behaviors as partly reflecting an organizational culture that had emerged around AOD and that encouraged and tolerated their presence.

Other examples of workplace drinking exist as well. Mangione and colleagues (1999) reported a large-scale survey of drinking in a sample of corporations and identified microcultures that encourage damaging and costly on-the-job drinking and tolerance of hangovers.

Sonnenstuhl (1996) described a pathological drinking culture that developed over nearly a century and that encouraged heavy and dangerous on- and off-the-job drinking among miners in New York City known as Sandhogs. However, Sonnenstuhl’s work is unique in that he documented the introduction of a “sobriety culture” among the Sandhogs through the emergence and on-the-job presence of coworkers who were recovering from alcoholism. The sobriety culture apparently tempered the excesses of the heavy drinking culture and created behavioral alternatives for those who did not want to drink heavily.

In a study that is uniquely valuable in substantiating the importance of organizational culture in preventing alcohol problems among employees, Ames and colleagues (2000) compared two work settings with distinctly different managerial cultures. One setting had a traditional hierarchical U.S. management design and the other was based on a Japanese management model transplanted to the United States. Although overall alcohol consumption rates in both populations were similar, the traditional management design was associated with more permissive norms regarding drinking before or during work shifts (including breaks) and higher workplace drinking rates. By contrast, the transplant management design was associated with greater enforcement of alcohol policies, which, in turn, predicted more conservative drinking norms and lower alcohol availability at work. Qualitative research clearly indicated that the transplant design facilitated the social control of alcohol problems whereas the traditional design appeared to undermine such control.

Beattie and colleagues (1992) developed and partially validated an instrument they titled “Your Workplace” (YWP), which can be used in job sites to measure the extent to which the workplace culture encourages drinking. Subsequent analysis of YWP found a strong and positive correlation between tolerance and encouragement of drinking by the workplace culture and clients’ levels of alcohol involvement (Rice et al. 1997).

Developing interventions that address problematic workplace cultures is challenging. Some researchers suggest that employees should face increasingly severe punishment for repeated on-the-job AOD use as a consequence of work-place rule violations. Mangione and colleagues (1999) speculate that health promotion and wellness programming may curb risky drinking practices.

## Conclusion

There is minimal current or recent research on the utility of EAPs and other mechanisms for addressing employed persons’ alcohol problems, as can be established from searching the National Institutes of Health database on funded research. Consequently, the research bases that have supported particular interventions in the past are dated and their application in today’s workplace may be challenged. This apparent lack of demand for such research may suggest that attention to workplace AOD abuse through these mechanisms may be declining (Roman in press).

There may be parallels in successfully addressing alcohol problems in the workplace and in primary and specialty medical care settings. The workplace domain and the medical care domain have the following in common: a great deal of preventive potential, the challenge of strongly competing goals within the domain, and problems of access for conducting research that meets scientific standards. Research over the past decade suggests that relatively modest investments in infrastructure can produce significant results in terms of physicians’ attention to alcohol problems (Fleming et al. 2000, 2002). An unspecified amount of such intervention and treatment occurs under the auspices of private physicians, but its quality remains unknown without intrusive monitoring. The significant extent of AOD abuse treatment and psychiatric care in nonspecialty hospitals has been documented, but this research did not include evidence about the nature or quality of care (Kiesler and Simpkins 1993).

Several additional specific parallels between primary medical care and workplace-based interventions highlight problems relating to AOD abuse research and practice. First, primary care settings and workplaces are both diverse and thus are not conducive to simple data collection methods. Second, the structure and content of intervention and treatment that occur in primary medical care and in workplace settings are highly variable. Third, the extent of such intervention is voluntary for both primary care physicians and employers. Fourth, in most primary medical care settings and in most workplaces, attention to alcohol problems is not a high priority goal. Fifth, as in the workplace, alcohol problems often become evident in the course of primary medical care, and the potential for intervention is great, especially given the extent to which this high-risk population seeks primary medical care as compared with specialty care. Finally, as in the work-place, there is very little research on the efficacy of the service delivery that occurs in these settings.

Beyond these issues, several other barriers exist that make it difficult to implement prevention programming directed at workplace AOD abuse. Employers’ resistance to workplace prevention stem from the following issues:

- Perceptions that data may uncover their liability for exacerbating AOD use and abuse
- Concern that alcohol specialists do not understand the workplace and would introduce interventions that are impractical and costly
- Lack of direct connections between alcohol problem interventions and workplace goals, with the connotation that reducing alcohol problems benefits the individual and the public good rather than the employer
- Problematic research access as a result of the sheer amount of time required to collect data from employees in active workplaces and the disruptions that research can cause (Roman and Baker 2001).

Thus there can be little doubt of the need for additional research focused on the workplace and alcohol issues. Data are needed to link the findings of studies that identify factors in the workplace related to problem drinking with interventions that are acceptable to employers. Data are also needed on the efficacy of specific work-place practices that have been adopted and that are targeted at alcohol-related issues. Finally, data are needed on how to sustain the workplace’s attention to employee alcohol issues in light of the competition of other goals and the intervention barriers unique to the workplace setting.
